# Survival and Durability of Minimally Invasive Mitral Valve Repair: Insights from Different Repair Techniques

**DOI:** 10.3390/medsci12030046

**Published:** 2024-09-02

**Authors:** Alessandra Iaccarino, Ilaria Giambuzzi, Denise Galbiati, Enea Cuko, Ginevra Droandi, Sara Forcina, Eraldo Kushta, Alessio Basciu, Alessandro Barbone, Andrea Fumero, Lucia Torracca

**Affiliations:** 1 Cardiovascular Department, UO of Cardiac Surgery of IRCCS Humanitas Research Hospital, 20089 Rozzano, Italyandrea.fumero@humanitas.it (A.F.);; 2 IRCCS Foundation Hospital San Matteo, University of Pavia, 27100 Pavia, Italy; eraldokushta7@gmail.com

**Keywords:** mitral valve repair, mini-thoracotomy mitral valve repair, minimally invasive mitral valve repair, edge-to-edge, quadrangular resection, artificial chordae

## Abstract

This study evaluates the long-term outcomes of minimally invasive mitral valve repair (MIMVR) in patients with degenerative mitral regurgitation, focusing on survival, mitral valve repair failure, and re-operation rates. A cohort of patients undergoing three primary repair techniques—quadrangular resection, edge-to-edge repair, and artificial chordae implantation—was analyzed using time-to-event methods. The overall survival rates at 1, 10, and 20 years were high and comparable among the techniques, indicating effective long-term benefits of MIMVR. However, freedom from recurrence of moderate mitral regurgitation (MR) ≥ 2 was significantly higher in the quadrangular resection and edge-to-edge groups compared to the artificial chordae group. No significant differences were observed for recurrent MR ≥ 3. Re-operation rates were low and similar across all techniques, underscoring the durability of MIMVR. Pre-discharge residual MR ≥ 2 was identified as a strong predictor of long-term repair failure. These findings confirm the effectiveness of MIMVR, with all techniques demonstrating excellent long-term survival and durability.

## 1. Introduction

Mitral valve regurgitation (MR) is a common valvular heart disease that significantly impacts patient morbidity and mortality. Mitral valve repair (MVP) is the standard of care for patients with degenerative MR. Minimally invasive mitral valve repair (MIMVR) has evolved over the years, and it permits the use of all the various techniques normally employed also through sternotomy [1].

MIMVR has gained popularity due to its potential benefits, such as reduced postoperative pain, shorter hospital stays, and quicker recovery times compared to conventional sternotomy. However, also the choice of surgical technique can influence intra-operative and postoperative outcomes, including cardiopulmonary bypass (CPB) and aortic cross-clamp times, as well as long-term valve function and survival rates. The feasibility of MIMVR in treating degenerative mitral disease has long been recognized. Many centers have incorporated this technique as a standard procedure for patients who do not have anatomical or clinical contraindications. The high success rates of modern mitral repair are largely due to the consistent achievement of long-lasting anatomical and functional valve repairs. Among the various techniques for mitral valve repair, certain methods have proven to be the most reliable and widely used.

The resection technique, an anatomical reconstruction pioneered by Carpentier, has been considered the gold standard since 1983 due to its excellent long-term results [2]. However, this technique has been associated with issues such as impaired left ventricular function, reduced annular area, decreased valve mobility, and increased tissue stress [3].

Tirone David introduced the neo-chordal technique using polytetrafluoroethylene (PTFE) sutures. This method restores the valve and ventricle geometry and movement, provides a large orifice area, maximizes the coaptation line, and reduces tissue stress. It quickly became a viable alternative to resection, also yielding excellent long-term results [4]. The introduction of artificial chordae opened the debate “Resect or Respect”, arguing which technique was better.

Both techniques have limitations in MIMVR: resection is time-consuming, and determining the correct length of neo-chordae is challenging. In parallel, Mohr and von Oppell developed the “Leipzig Loop Technique” to simplify MIMVR by using premeasured chordae [5].

Since posterior mitral leaflet prolapse is the most common and relatively straightforward to repair, studies report excellent long-term outcomes using either the respect or resect technique [6,7]. In contrast, repairing anterior mitral leaflet and bi-leaflet prolapses is more complex and typically yields less favorable results.

In 1991, Ottavio Alfieri introduced the “edge-to-edge” technique for repairing mitral regurgitation in such cases. By applying a suture at the site of the regurgitant jet between the anterior and posterior leaflets (usually A2 and P2), this method creates a “double-orifice” valve, eliminating residual prolapse [8]. This study retrospectively evaluates the pre-operative characteristics, surgical techniques, and peri-operative outcomes of patients who underwent MIMVR. The aim is to characterize the impact of the main techniques (quadrangular resection, edge-to-edge and artificial chordae) on long-term follow-up in terms of mortality and mitral valve repair failure.

## 2. Materials and Methods

### 2.1. Study Population

This is a retrospective study focusing on patients who underwent mitral valve repair via mini-thoracotomy between 1999 and 2023, performed by a single experienced surgeon. Only patients diagnosed with degenerative MR were included. The study was conducted in accordance with the declaration of Helsinki, and the local committee (CET Lombardia 5) granted authorization because of the retrospective nature of the study (retrospective study n°35/24).

Only patients undergoing mitral valve repair via mini-thoracotomy were included. Patients younger than 18 years old with secondary mitral regurgitation were excluded.

Mitral valve pathology was confirmed through transthoracic (TTE) and trans-esophageal (TEE) echocardiograms.

Patients were categorized into five groups based on the surgical technique used for mitral valve repair: quadrangular resection, edge-to-edge, artificial chordae, isolated annuloplasty, and a combination of techniques. The main objective was to analyze long-term survival and long-term MVR failure. Mitral valve repair failure included patients with recurrent MR (≥2, ≥3, and REDO surgery on mitral valve).

### 2.2. Surgical Technique

A small (5–7 cm) right anterolateral mini-thoracotomy was performed in the 3rd or 4th intercostal space. Cardiopulmonary bypass (CPB) was established through femoral arterial and venous cannulation. Endoscopic vision was facilitated using a 30° camera and full HD system. The aorta was cross-clamped either by a Chitwood or Cignet malleable clamp through the thoracotomy, and myocardial protection was achieved via antegrade delivery of cold crystalloid cardioplegia. The left atrium was opened through the interatrial groove, and a left atrial retractor was used to expose the mitral valve (MV). Throughout the surgical procedure, CO_2_ was actively insufflated into the chest cavity; intra-cardiac air removal was achieved by active ventricular and aortic root suction. Various surgical techniques were employed during the study period, with quadrangular resection, edge-to-edge, and artificial neochordae being the primary ones. A prosthetic ring was always implanted. TEE was routinely performed at the end of CPB to evaluate the presence of residual MR. A comprehensive echocardiographic evaluation was conducted once the patient regained physical autonomy, typically on the 4th postoperative day.

### 2.3. Follow-Up

Long-term follow-up was completed in 98.58% of cases. Mitral valve function was assessed based on the most recent echocardiographic examination available, either during check-ups at our institution or with the referring cardiologist. Follow-up data were gathered through telephone contact with the patient or family members.

### 2.4. Statistical Analysis

Data were presented as the mean ± standard deviation or as median and interquartile range (IQR). The Shapiro–Wilk test determined whether the distribution was normal or non-normal. Categorical data were presented as absolute numbers and frequencies and compared using the χ^2^ test, with the Holm test applied when necessary. Comparison of continuous data (expressed as median and IQR) was performed using the Kruskal–Wallis test, with Bonferroni correction applied as needed. The simple imputation method was applied to missing values. The significance level in hypothesis testing was set to 0.05.

Time-dependent variables as survival and mitral valve failure stratified by type of repair techniques were plotted with Kaplan–Meier curves and compared with the log-rank test. Mitral valve repair failure was defined as recurrent MR ≥ 2+ or MR ≥ 3+ and mitral valve re-intervention. A Cox proportional hazards model was used to identify predictors of mitral failure. The proportional hazards assumption of the Cox model was assessed using the Schoenfeld residual test. Collinearity among variables was checked through the variance inflation factor (VIF) for each predictor variable. In the Cox proportional hazard models, the reference category for mitral techniques was quadrangular resection, for mitral lesion was posterior prolapse, for sex it was male, and for annular prosthetics it was incomplete rings.

In the time-to-event analysis, due to the limited number of patients in the isolated annuloplasty and combination of techniques groups, only patients who underwent quadrangular resection, edge-to-edge, or artificial chordae implantation were included.

Statistical analyses were conducted using R-Studio software (RStudio 2024.04.02).

## 3. Results

### 3.1. Pre-Operative Characteristics, Surgical Techniques and Peri-Operative Outcomes

Between December 1999 and December 2023, a total of 538 patients underwent MIMVR. They were divided into 5 groups depending on the type of mitral repair technique they received: quadrangular resection (163 patients, 30.3%), edge-to-edge—either central or paracommisural—(192 patients, 35.69%), artificial neochordae (139 patients, 25.83%), a combination of the three (17 patients, 3.16%), and isolated annuloplasty (27 patients, 5.02%). Pre-operative characteristics are listed in Table 1.

There was a significant difference in age among groups. A Bonferroni correction was applied, and patients who received edge-to-edge were significantly younger than the ones in the other groups, and the patients with artificial chordae were older than patients with resection. Female patients were significantly more common in the edge-to-edge group at the Holm test. Moreover, the lowest EF was in the patients treated with edge-to-edge. Intra-operative and post-operative characteristics are listed in Table 2.

Regarding surgery, only 5 (0.9%) patients were converted to sternotomy because of inappropriate exposure and uncontrollable bleeding. Only 11 (2.05%) patients had a second aortic cross-clamping to review residual MR. CPB time and aortic cross-clamping times were significantly different among groups. At Bonferroni analysis, the significant differences were among patients in the edge-to-edge group when compared to chordae and combination, and also showed a significantly shorter aortic cross-clamping than resection. Artificial chordae group had also longer CPB and aortic cross-clamping times than resection. The patients who received the ring alone did not show any significant difference with any other groups regarding both CPB and aortic cross-clamping time. Patients in the Ring group showed a higher incidence (without reaching statistical significance) of tricuspid valve surgery, which might partially explain the trend of longer surgical times.

The overall rates of post-operative complications were low (Table 2), and no difference was found between groups. No intra-hospital death was recorded. At discharge, 21 (3.9%) patients had a moderate residual MR and 1 patient (0.19%) severe residual MR (who underwent early mitral valve replacement).

### 3.2. Follow-Up

Median follow-up time was 6.56 years [2.04–9.61] and was 98.58% completed.

At follow-up, there were 5 (1%) deaths (without differences among groups, *p*-value 0.277). A total of 11 (2.1%) re-operations for mitral valve disease (among groups, *p*-value 0.219). Recurrent MR ≥ 2 at echocardiogram was found in 44 (8.4%) patients (*p*-value 0.897), while recurrent MR ≥ 3 was found in 25 (4.8%) patients (*p*-value 0.158).

In the time-to-event survival analysis, only patients in the resection, edge-to-edge, and chordae were included, as they were the most commonly used procedures. Therefore, a total of 494 patients were included in the analysis. Among the group of patients, there were 4 (0.8%) deaths at follow-up, 39 (7.89%) patients with MR ≥ 2 and 20 (4%) patients with MR ≥ 3, and 9 (1.82%) patients underwent re-intervention. Mean follow-up time in the resection group, edge-to-edge group, and chordae group was, respectively, 7.72 ± 5.60 years, 7.08 ± 5.73 years, and 4.19 ± 3.39 years.

**Figure 1:**
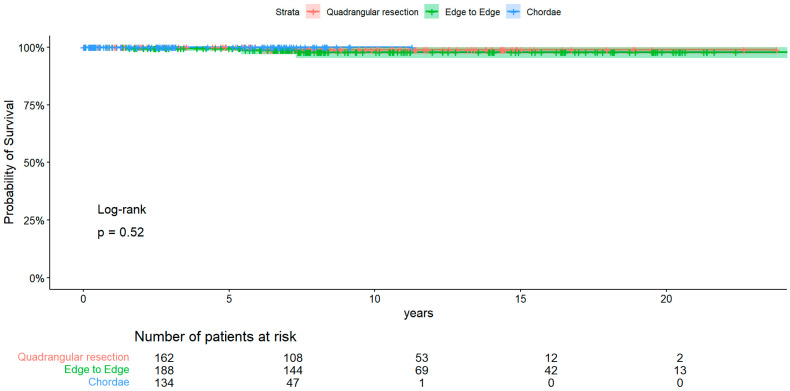
KM of overall death.

Regarding overall death [Figure 1], there were no differences among the group at log-rank analysis (*p*-value = 0.52). Freedom from death at 1, 10, and 20 years in the resection group was, respectively, 100% (CI 95% 1–1, reflecting absence of events and censoring), 99% (CI 95% 0.969–1), stable also at 20 years. Regarding the edge-to-edge group, freedom from death at 1, 10, and 20 years was, respectively, 100% (CI 95% 1–1, reflecting absence or events and censoring), 97.8% (CI 95% 0.953–1) stable at 20 years. The chordae group had a lower follow-up time, and survival was 100% up to 10 years.

**Figure 2:**
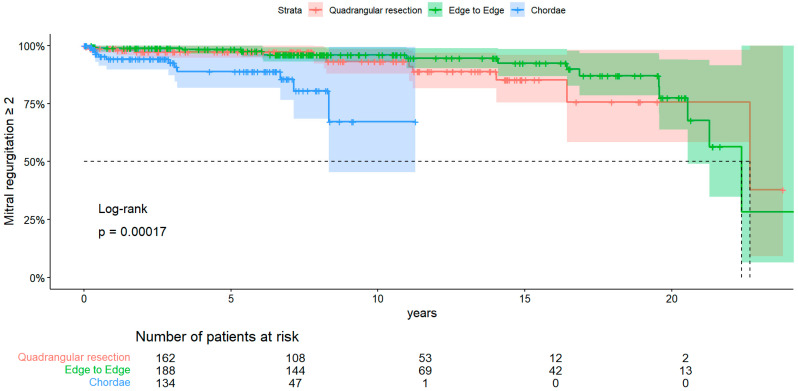
KM of recurrent MR ≥ 2.

Regarding recurrent MR ≥ 2 (Figure 2), a significant difference among groups was found (*p*-value < 0.001). Freedom from MR ≥ 2 at 1, 10, and 20 years in the resection group were, respectively, 98.7% (CI 95% 0.97–1), 93.1% (CI 95% 0.88–0.98), and 75.7% (CI 95% 0.58–0.98). In the edge-to-edge group, freedom from MR ≥ 2 at 1, 10, and 20 years was, respectively, 98.9% (CI 95% 0.97–1), 96.1% (CI 95% 0.93–0.99), and 77.4% (CI 95% 0.64–0.94). In the chordae group, freedom from MR ≥ 2 at 1, 5, and 10 was, respectively, 94.2% (CI 95% 0.89–0.99), 88.8% (CI 95% 0.82–0.96), and 67.1% (CI 95% 0.45–0.99).

A multivariate Cox regression analysis was performed, and results are shown in Table 3. Collinearity was performed, and the VIF values were as follows: Pre-discharge MR ≥ 2 (VIF = 1.005), edge-to-edge (GVIF = 1.003), and artificial chordae (GVIF = 1.003).

**Figure 3:**
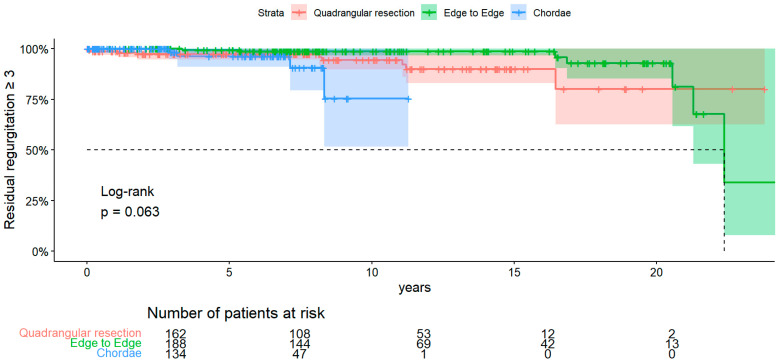
KM of recurrent MR ≥ 3.

Regarding recurrent MR ≥ 3 (Figure 3), no significant difference among groups was found (*p*-value = 0.063). Freedom from MR ≥ 3 at 1, 10, and 20 years in the resection group was, respectively, 98.7% (CI 95% 0.97–1), 94.4% (CI 95% 0.89–0.99), and 80% (CI 95% 0.63–1). In the edge-to-edge group, freedom from MR ≥ 3 at 1, 10, and 20 years was, respectively, 100% (CI 95% 1–1), 98.6% (CI 95% 0.97–1), and 92.9% (CI 95% 0.85–1). In the chordae group, freedom from MR ≥ 3 at 1, 5, and 10 was, respectively, 100% (CI 95% 1–1), 96.2% (CI 95% 0.91–1), and 75.5% (CI 95% 0.51–1).

A Cox regression univariate analysis was performed, and results are shown in Table 4. Only MR ≥ 2 was a significant predictor.

## 4. Discussion

The findings of this retrospective study shed light on the outcomes and prognostic factors associated with MIMVR procedures. Our analysis encompasses pre-operative characteristics, surgical techniques, peri-operative outcomes, and long-term follow-up data, providing valuable insights into the management of degenerative MR.

In terms of outcomes, recent studies demonstrate that MIMVR, compared with the conventional sternotomy approach, can be performed without significant differences in mortality and long-term survival [9,10,11,12]. Despite slightly longer CPB and cross-clamp times [13,14], this approach leads to comparable rates of peri-operative mortality, long-term survival, and freedom from re-operation [9,12,15].

The study cohort, comprising 538 patients who underwent MIMVR between 1999 and 2023, was stratified into five groups based on the type of mitral repair technique utilized. Significant variations in age distribution were observed among these groups, with younger patients more commonly undergoing edge-to-edge repair and artificial chordae implantation. Additionally, female patients were more prevalent in the edge-to-edge group.

### 4.1. Peri-Operative Outcomes

Current literature shows that patients undergoing MIMVR have a similar or even lower incidence of post-operative complications than those of patients undergoing median sternotomy [8,10,11]. A higher rate of stroke after a minimally invasive approach as a consequence of peripheral cannulation or retrograde perfusion might be considered a theoretical disadvantage of this approach [12]. Anyway, some authors disprove these findings given that many studies have shown that there is no significant difference in the incidence of stroke in the two types of surgical approach [7,11,13]. The incidence of stroke in the literature ranges from 0.5% to 2.1% [16]. Specific protocols have been developed to ensure that all patients scheduled for MIMVR undergo a pre-operative CT scan. This is to guarantee safe remote access perfusion and to rule out those who have contraindications for arterial perfusion via the femoral artery. Due to the high standardization of the technique, we can now achieve thorough de-airing through the ascending aorta and CO_2_ flushing of the right hemithorax. In our series, these results have been confirmed, as the incidence of stroke was very low (0.4%).

Modi et al. reported a systematic meta-analysis of six studies showing similar stroke incidence between minimally invasive and conventional approaches [17]. Longer times of cross-clamp and CPB do not influence early mortality. Conversely, patients treated with a minimally invasive approach less frequently develop renal failure requiring dialysis treatment and show better early survival [9,12,15,18,19,20]. The frequency of bleeding, respiratory failure, and wound infections is comparable to that of the traditional sternotomy approach [9,12,15,18].

Intra-operative and post-operative variables, including conversion to sternotomy, aortic cross-clamping, and cardiopulmonary bypass (CPB) times, were assessed across the different repair techniques. In line with the published literature, edge-to-edge repair demonstrated shorter CPB and aortic cross-clamping times compared to other techniques, highlighting its potential advantages in terms of operative efficiency [21].

Moreover, it is important to underline the low rate of conversion to sternotomy, which was only 0.9%. Many studies examined the influence of MIMVR on the success of mitral valve repair, demonstrating similar or superior repair success rates compared to those of the conventional approach [10,13,16,19]. In our series, residual MR = 2 was present in 14 patients (2.6%), and only in one case (0.2%) the residual MR was severe and required early replacement. Moreover, no intra-hospital death was recorded over a period of 25 years. These results reflect that MIMVR yields the same safety profile as sternotomy, regardless of the type of techniques employed to repair the valve.

### 4.2. Long-Term Follow-Up and Survival Analysis

The durability of mitral repair is the most crucial predictor of long-term outcomes, influenced by both the repair technique and the location of the primary pathology. Kinza Iqbal et al. [22] conducted a meta-analysis to compare long-term outcomes based on different types of mitral valve lesions, analyzing data from ten articles. This study included 9319 participants, with 2727 undergoing anterior/bilateral mitral valve repair and 6592 undergoing posterior MVP. The follow-up period for assessing long-term outcomes ranged from 5 to 15 years. The study found no significant difference in long-term survival or freedom from moderate-to-severe mitral regurgitation and reoperation between anterior/bi-leaflet and posterior leaflet repairs for mitral valve degenerative disease. The choice and quality of repair significantly impact outcomes, depending largely on the surgeon’s ability to select the appropriate technique for each case.

In the extensive range of retrospective studies that emphasize long-term follow-up, distinctions between standard and MIMVR are often unclear. Conversely, studies on minimally invasive surgery populations tend to focus on shorter-term outcomes [23]. L. Polzl et al. [24] evaluated a consecutive series of patients with Barlow’s disease undergoing minimally invasive MV surgery from 2001 to 2020 (*n* = 246). Patients were grouped by surgical technique (isolated annuloplasty, use of artificial chordae, or leaflet resection). No significant differences were found between the techniques regarding primary outcomes, including MVARC-event-free survival (mortality, reoperation due to repair failure, or recurrence of severe MR within 5 years). Operative success, defined as primary mitral repair without conversion to valve replacement or major thoracic incisions, no residual MR > mild, and no need for reoperation within the first 30 days, was achieved in 93.5%. Kaplan–Meier estimates showed no significant difference in 5-year mortality or freedom from reoperation among the techniques (log-rank: *p*-value 0.419 for mortality, 0.205 for reoperation and mortality). MVARC-event-free survival did not differ significantly between groups (2.9% vs. 4.6% vs. 0%; *p*-value 0.244).

Studies on the long-term outcomes of mitral valve repair have previously been conducted [25,26]. De Bonis et al. [25], for example, reported data on 139 patients undergoing mitral valve repair with the edge-to-edge technique, with a mean follow-up of 11.5 years. At 17 years, survival was 72.4% ± 7.89%, freedom from cardiac death was 90.8% ± 4.77%, freedom from re-operation was 89.6% ± 2.74%, and freedom from MR ≥ 3+ was 80.2% ± 5.86%.

Our median follow-up duration of 6.56 years revealed low mortality rates (1%) and a modest incidence of re-operations for mitral valve disease (2.1%) across our study cohort. The longest follow-up time was 24 years.

Survival analysis demonstrated similar long-term outcomes among the different repair techniques, with no significant differences in overall mortality rates observed. However, significant disparities were noted in freedom from moderate MR (≥2), as artificial chordae had a higher risk of MR ≥ 2 at follow-up. Nevertheless, there was no difference among groups in long-term MR ≥ 3. As expected, the only important predictor that remained significant was pre-discharge moderate MR (≥2) as a strong predictor of adverse outcomes, emphasizing the importance of early post-operative assessment and management.

These results add new insight to the debate on the “resect versus respect” approach. Artificial chordae exhibit a higher rate of recurrent MR ≥ 2 but not for recurrent MR ≥ 3. This observation suggests that technical challenges, such as early and late chordal rupture, incorrect placement, and histopathological phenomena (chordae calcification/no endothelization [27,28]), may play a significant role in MR recurrence. Nevertheless, further studies are needed to clarify if positive remodeling of the left ventricle [29,30] subsequent to mitral valve repair has more impact on artificial chordae [31,32]. Despite being the most respectful technique on the mitral anatomy, the length of the artificial chordae does not adapt as the left ventricle goes back to its original dimensions [33].

Nevertheless, even if the recurrent MR is moderate in patients with artificial chordae, it does not seem to significantly progress towards a meaningful MR.

A recent meta-analysis by Tulio Caldonazo et al. [34] showed that there were no differences between resection and artificial chordae regarding MR recurrence, but the authors do not specify the degree. It is indeed known, and in line with our results, that a pre-discharge MR ≥ 2 is the most important risk factor for recurrence. Nevertheless, in patients with artificial chordae, MR seems to stabilize to a moderate degree during follow-up.

### 4.3. Study Limitations

The primary limitation of our study is its retrospective design, which means that the results may be affected by factors such as patient selection, the operator’s experience, and the techniques employed over the extensive time period examined. Potential biases related to changes in surgical techniques and the surgeon’s experience over time cannot be ruled out. Additionally, comprehensive echocardiographic data collection during follow-up was hindered because these exams were only partially performed at our institution, with many patients being followed up at external centers. Moreover, limited data on left ventricle function and dimensions, or valve failure mechanisms, were available at follow-up.

## 5. Conclusions

This study provides valuable insights into the outcomes of MIMVR (MIMVR) for patients with degenerative MR, focusing on long-term survival and mitral valve repair failure. The study confirms that MIMVR is a viable and effective option for treating degenerative MR, despite the chosen technique of repair, with excellent long-term survival and low rates of repair failure and re-operation.

## Figures and Tables

**Table 1:** Pre-operative characteristics.

Variable	Overall	Resection	Edge-To-Edge	Chordae	Ring	Combination	*p*-Value
**TOTAL**	538	163	192	139	27	17	
FEMALE	188 (34.9)	41 (25.2)	94 (49.0)	34 (24.5)	14 (51.9)	5 (29.4)	<0.001
AGE (yrs)	48.39 ± 12.42	49.53 ± 10.85	42.43 ± 12.93	53.76 ± 9.93	51.87 ± 14.7	53.60 ± 7.79	<0.001
NYHA > 3	69 (12.8)	29 (17.8)	25 (13.0)	10 (7.2)	3 (11.1)	2 (11.8)	0.106
**NYHA**							0.149
1	272 (50.6)	65 (39.9)	104 (54.2)	80 (57.6)	12 (44.4)	11 (64.7)	
2	197 (36.6)	69 (42.3)	63 (32.8)	49 (35.3)	12 (44.4)	4 (23.5)	
3	67 (12.5)	28 (17.2)	24 (12.5)	10 (7.2)	3 (11.1)	2 (11.8)	
4	2 (0.4)	1 (0.6)	1 (0.5)	0 (0.0)	0 (0.0)	0 (0.0)	
REDO	1 (0.2)	0 (0.0)	1 (0.5)	0 (0.0)	0 (0.0)	0 (0.0)	0.771
EF (%)	63 [60–68]	65 [60–69]	60 [58–65]	64 [60–68]	62 [57.5–64]	62.5 [60–68.5]	0.02
**RHYTHM**							0.066
SR	495 (92.0)	150 (92.0)	172 (89.6)	136 (97.8)	23 (85.2)	14 (82.4)	
AF	41 (7.6)	13 (8.0)	19 (9.9)	2 (1.4)	4 (14.8)	3 (17.6)	
PM	2 (0.4)	0	1(0.5)	1 (0.7)	0	0	
**PROLAPSE**							
PL	333 (61.9)	162 (99.4)	22 (11.5)	125 (89.9)	13 (48.1)	11 (64.7)	<0.001
AL	52 (9.7)	0 (0.0)	36 (18.8)	8 (5.8)	5 (18.5)	3 (17.6)	<0.001
BL	157 (29.2)	1 (0.6)	134 (69.8)	10 (7.2)	5 (18.5)	7 (41.2)	<0.001

REDO: re-intervention, EF: ejection fraction; SR: sinus rhythm; AF: atrial fibrillation; PM: pacemaker; PL: posterior leaflet; AL: anterior leaflet; BL: bi-leaflet. Continuous variables are presented as “median [IQR]” or “mean ± SD”, categorical variables are presented as absolute values and percentages *n* (%).

**Table 2:** Intra-operative and post-operative characteristics.

Variable	Overall	Resection	Edgetoedge	Chordae	Ring	Combination	*p*-Value
	538	163	192	139	27	17	
**Intra-Operative Data**							
CPB time (min)	117 [95.2–143]	104 [90–124]	104 [84–129]	134 [116–156]	120 [95–151]	159 [126–194]	<0.001
Cross-clamping time (min)	85 [70–107]	81 [69–98.8]	74 [62–93]	100 [83–116]	93 [68–117]	116 [91.2–142]	<0.001
Tricuspid valve surgery	125 (23.2)	32 (19.6)	40 (20.8)	39 (28.1)	9 (33.3)	5 (29.4)	0.237
**Post-Operative Complication**							
PM implant	2 (0.4)	0	2 (1)	0	0	0	0.460
IABP	23 (4.3)	2 (1.2)	8 (4.2)	10 (7.2)	1 (3.7)	2 (11.8)	0.062
Transfusion	72 (13.4)	18 (11)	26(13.5)	18(12.9)	4 (14.8)	6 (35.3)	0.069
Respiratory failure	7 (1.3)	2 (1.2)	3 (1.6)	1 (0.7)	0	1 (5.9)	0.461
Surgical revision	7 (1.3)	0	3 (1.6)	2 (1.4)	2 (7.4)	0	0.035
AF	88 (16.4)	22 (13.5)	32 (16.7)	24 (17.3)	6 (22.2)	4 (23.5)	0.664
AMI	5 (0.9)	2 (1.2)	2 (1)	1 (0.7)	0	0	0.956
STROKE	2 (0.4)	0	1 (0.5)	0	0	1 (5.9)	0.004
Intra-hospital mortality	0	0	0	0	0	0	0
Surgical wound problems	6 (1.1)	1 (0.6)	3 (1.6)	2 (1.4)	0	0	0.853
Pericardial effusion	11 (2)	2 (1.2)	5 (2.6)	3 (2.2)	1 (3.7)	0	0.813
**Post-op MR**							0.362
0	413 (76.8)	120 (73.6)	153 (79.7)	105 (75.5)	20 (74.1)	15 (88.2)	
1	110 (20.4)	37 (22.7)	32 (16.7)	34 (24.5)	6 (22.2)	1 (5.9)	
2	14 (2.6)	5 (3.1)	7 (3.6)	0 (0.0)	1 (3.7)	1 (5.9)	
3	0	0	0	0	0	0	
4	1 (0.2)	1 (0.6)	0 (0.0)	0 (0.0)	0 (0.0)	0 (0.0)	
Post-op MR ≥ 2	15 (2.8)	7 (4.3)	9 (4.7)	4 (2.9)	1 (3.7)	1 (5.9)	0.930

AF: atrial fibrillation, PM: pacemaker, MR: mitral regurgitation, AMI: acute myocardial infarction, Surgical Revision: revision for bleeding.

**Table 3:** Cox regression analysis for MR ≥ 2.

Variable	Univariate			Multivariate		
	HR	CI	*p*-Value	HR	CI	*p*-Value
Pre-discharge MR ≥ 2	7.25	3.523–14.922	<0.001	10.17	4.52–22.86	<0.001
AL	1.08	0.4256–2.7554	0.866			
BL	0.51	0.2446–1.1011	0.08			
Sex	1.06	0.4877–2.3229	0.856			
NYHA > 2	1.16	0.6888–1.9513	0.724			
TVR	1.322	0.6026–2.9012	0.486			
EtE	0.73	0.329–1.611	0.433	0.90	0.4073–2.01	0.803
ArtCh	4.02	1.647–9.854	0.002	6.28	2.39–16.48	<0.001

AL: anterior leaflet, BL: bi-leaflet, EtE: edge-to-edge, TVR: tricuspid valve repair, ArtCh: artificial chordae.

**Table 4:** Cox regression analysis for MR ≥ 3.

Variable	Univariate		
	HR	CI	*p*–Value
Pre-discharge MR ≥ 2	11.33	4.38–29.34	<0.01
AL	0.658	0.14–3.09	0.569
BL	0.604	−0.22–1.63	0.317
Sex	1.49	0.60–3.70	0.391
NYHA > 2	0.91	0.27–3.16	0.89
TVR	1.79	0.640–5.028	0.264
EtE	0.4349	0.156–1.212	0.111
ArtCh	2.04	−0.586–2.017	0.280

AL: anterior leaflet, BL: bi-leaflet, EtE: edge-to-edge, TVR: tricuspid valve repair, ArtCh: artificial chordae.

## Data Availability

Raw data are available upon request to the corresponding author.
